# Sportsmen and Charity

**Published:** 1920-06-12

**Authors:** 


### Sportsmen and Charity.

### A LEICESTER LEAD.

The columns of The Hospital have borne witness to many helpful associations between sportsmen, sporting institutions, and hospitals. In pre-war days reference was made to the expressed intention of the Leicester (Rugby) Football Club to be permanently associated with the Leicester Royal Infirmary by means of an endowed bed. The War postponed for a time the Annual Charity Match, arranged by the Club, but at the close of the past season the match was renewed; a strong London team being brought down by Mr. G. H. Harnett, of the Rugby Union. In consequence of the match a sum of £443 was raised, and, incidentally, the endowment of the first bed by the football club was completed. The Club has informed the Infirmary that the endowment of a second bed will proceed during the coming season.

The President of the Club is Mr. H. W. Salmon and the Honorary Secretary Mr. T. H. Crumbie, both of whom are life governors of the Infirmary and have figured in many efforts by which the Institution has been considerably helped. The Infirmary Committee was also cheered by learning, at the close of the season, that the Leicester City Football Club had also arranged a Benefit Match; the first, the Committee hoped, of a series of permanent fixtures, and the new Treasurer of the Institution, Mr. Duncan Henderson, J.P., had the pleasure of receiving from Mr. W. A. Jennings, the President, and Mr. Peter Hodge, the Secretary and Manager, the sum of £223. The City Football Committee hopes also by Charity Matches to accumulate contributions until the sum required for the endowment of a bed in the Institution is reached.

